# Radiomics in medical imaging—“how-to” guide and critical reflection

**DOI:** 10.1186/s13244-020-00887-2

**Published:** 2020-08-12

**Authors:** Janita E. van Timmeren, Davide Cester, Stephanie Tanadini-Lang, Hatem Alkadhi, Bettina Baessler

**Affiliations:** 1 Department of Radiation Oncology, University Hospital Zurich, University of Zurich, Raemistrasse 100, 8091 Zurich, Switzerland; 2 Institute of Diagnostic and Interventional Radiology, University Hospital Zurich, University of Zurich, Raemistrasse 100, 8091 Zurich, Switzerland

**Keywords:** Radiomics, Quantitative imaging biomarkers, Machine learning, Standardization, Robustness

## Abstract

Radiomics is a quantitative approach to medical imaging, which aims at enhancing the existing data available to clinicians by means of advanced mathematical analysis. Through mathematical extraction of the spatial distribution of signal intensities and pixel interrelationships, radiomics quantifies textural information by using analysis methods from the field of artificial intelligence. Various studies from different fields in imaging have been published so far, highlighting the potential of radiomics to enhance clinical decision-making. However, the field faces several important challenges, which are mainly caused by the various technical factors influencing the extracted radiomic features.

The aim of the present review is twofold: first, we present the typical workflow of a radiomics analysis and deliver a practical “how-to” guide for a typical radiomics analysis. Second, we discuss the current limitations of radiomics, suggest potential improvements, and summarize relevant literature on the subject.

## Key points

- Radiomics represents a method for the quantitative description of medical images.
- A step-by-step “how-to” guide is presented for radiomics analyses.
- Throughout the radiomics workflow, numerous factors influence radiomic features.
- Guidelines and quality checklists should be used to improve radiomics studies’ quality.
- Digital phantoms and open-source data help to improve the reproducibility of radiomics.

## Background

Like many other areas of human activity in the last decades, medicine has seen a constant increase in the digitalization of the information generated during clinical routine. As more medical data became available in digital format, new and always more sophisticated software was developed to analyze them. At the same time, the research on artificial intelligence (AI) has long reached a point where its methods and software tools have become not only powerful, but also accessible enough to leave the computer science departments and find applications in an increasing variety of domains. As a consequence, the recent years have witnessed a continuous increase of AI applications in the medical sector, aiming at facilitating repetitive tasks clinicians encounter in their daily clinical workflows and to support clinical decision-making.

The different techniques used in AI—i.e., mainly machine learning and deep learning algorithms—are especially useful when it comes to the emerging field of “big data”. Big data is defined as “*a term that describes large volumes of high velocity*, *complex and variable data that require advanced techniques and technologies to enable the capture*, *storage*, *distribution, management*, *and analysis of the information*.” 1 Due to the high amount of multi-dimensional information, techniques from the field of AI are needed to extract the desired information from these data.

In medicine, various ways to generate big data exist, including the widely known fields of genomics, proteomics, or metabolomics. Similar to these “omics” clusters, imaging has been used increasingly to generate a dedicated omics cluster itself called “radiomics”. Radiomics is a quantitative approach to medical imaging, which aims at enhancing the existing data available to clinicians by means of advanced, and sometimes non-intuitive mathematical analysis. The concept of radiomics, which has most broadly (but not exclusively) been applied in the field of oncology, is based on the assumption that biomedical images contain information of disease-specific processes [1] that are imperceptible by the human eye [2] and thus not accessible through traditional visual inspection of the generated images. Through mathematical extraction of the spatial distribution of signal intensities and pixel interrelationships, radiomics quantifies textural information [3, 4] by using analysis methods from the field of AI. In addition, visual appreciable differences in image intensity, shape, or texture can be quantified by means of radiomics, thus overcoming the subjective nature of image interpretation. Thus, radiomics does not imply any automation of the diagnostic processes, rather it provides existing ones with additional data.

Radiomics analysis can be performed on medical images from different modalities, allowing for an integrated cross-modality approach using the potential additive value of imaging information extracted, e.g., from magnetic resonance imaging (MRI), computed tomography (CT), and positron-emission-tomography (PET), instead of evaluating each modality by its own. However, the current state-of-the-art of the research still shows lack of stability and generalization, and the specific study conditions and the authors’ choices have still a great influence on the results.

In this work, we present the typical workflow of a radiomics analysis, discussing the current limitations of this approach, suggesting potential improvements, and commenting relevant literature on the subject.

## Radiomics–how to?

**Fig. 1:**
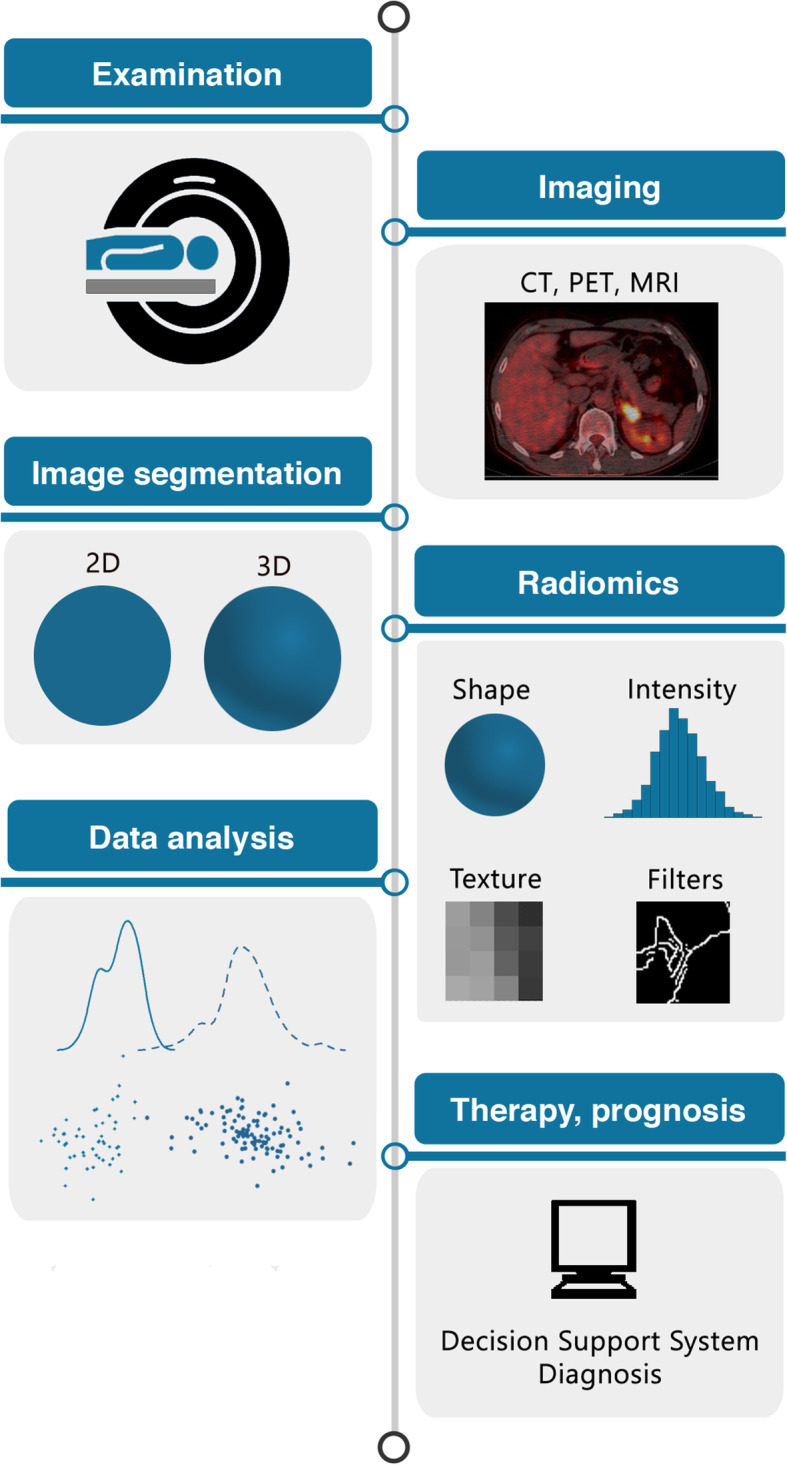
The radiomics workflow. Schematic illustration of the patient journey including image acquisition, analysis utilizing radiomics, and derived patient-specific therapy and prognosis. After image acquisition and segmentation, radiomic features are extracted. High-level statistical modeling involving machine learning is applied for disease classification, patient clustering, and individual risk stratification

The following section will give a practical advice on “how to do radiomics” by illustrating each of the required steps in the radiomics pipeline (illustrated in Fig. 1) and highlighting important points.

### Step 1: image segmentation

For any radiomics approach, delineation of the region of interest (ROI) in two-dimensional (2D) or of the volume of interest (VOI) in three-dimensional (3D) approaches is the crucial first step in the pipeline. ROIs/VOIs define the region in which radiomic features are calculated.

Image segmentation might be done manually, semi-automatically (using standard image segmentation algorithms such as region-growing or thresholding), or fully automatically (nowadays using deep learning algorithms). A variety of different software solutions—either open-source or commercial—are available, such as 3D Slicer 2 [5], MITK 3, ITK-SNAP 4, MeVisLab 5, LifEx 6, or ImageJ 7 [6], to name only some frequently used open-source tools. For reviews on various different tools for image segmentation, please refer to [7, 8].

Manual and semi-automated image segmentation (usually with manual correction) are the most often encountered methods but have several drawbacks. Firstly, manual segmentation is time-consuming – depending on how many images and datasets have to be segmented. Second, manual and semi-automated segmentation introduce a considerable observer-bias, and studies have shown that many radiomic features are not robust against intra- and inter-observer variations concerning ROI/VOI delineation [9]. Consequently, studies using manual or semi-automated image segmentation with manual correction should perform assessments of intra- and inter-observer reproducibility of the derived radiomic features and exclude non-reproducible features from further analyses.

Deep learning-based image segmentation (often using some sort of U-Net [10]) is rapidly emerging and many different algorithms have already been trained for image segmentation tasks of various organs (currently, most of them being useful for the segmentation of entire organs, but not for segmentation of dedicated tumor regions), several of them being published as open-source. Since recently, there are also several possibilities for integration of such algorithms in platforms like 3D Slicer or MITK. Automated image segmentation certainly is the best option, since it avoids intra- and inter-observer variability of radiomic features. However, generalizability of trained algorithms currently is a major limitation, and applying those algorithms on a different dataset often results in complete failure. Thus, further research has to be devoted to the development of robust and generalizable algorithms for automated image segmentation.

### Step 2: image processing

Image processing is located between the image segmentation and feature extraction step. It represents the attempt to homogenize images from which radiomic features will be extracted with respect to pixel spacing, grey-level intensities, bins of the grey-level histogram, and so forth. Preliminary results have shown that the test-retest robustness of radiomic features extracted largely depends on the image processing settings used [11–15]. In order to allow for reproducible research, it is therefore important to report each detail of the image processing step.

Several of the above-mentioned software platforms (namely, 3D Slicer and LifEx) have integrations for radiomics analyses. 3D Slicer has incorporated an installable plugin for the open-source pyRadiomics package [16] (which can otherwise be used within a solo Python framework), whereas LifEx is a stand-alone platform with integrated segmentation and texture analysis tools and a graphical user interface. The image processing step in the pyRadiomics package (which currently is one of the most commonly used packages for radiomics analyses) can be defined by writing a so-called parameter file (in a YAML or JSON structured text file). This parameter file can be loaded into 3D Slicer or be incorporated into a Python framework. Example parameter files for different modalities can be found in the pyRadiomics GitHub repository^8^.

*Interpolation to isotropic voxel spacing* is necessary for most texture feature sets to become rotationally invariant and to increase reproducibility between different datasets [17]. Currently, there is no clear recommendation whether upsampling or downsampling should be the preferred method. In addition, data from different modalities might need different approaches for image interpolation. CT, for example, usually delivers isotropic datasets, whereas MRI often delivers non-isotropic data with need for different approaches to interpolation. After applying interpolation algorithms to the image, the delineated ROI/VOI should also be interpolated. For a detailed description of image interpolation and different interpolation algorithms, please refer to [17].

*Range re-segmentation and intensity outlier filtering (normalization)* are performed to remove pixels/voxels from the segmented region that fall outside of a specified range of grey-levels [17]. Whereas range re-segmentation usually is required for CT and PET data (e.g., for excluding pixels/voxels of air or bone within a tumor ROI/VOI), range re-segmentation is not possible for data with arbitrary intensity units such as MRI. For MRI data, intensity outlier filtering is applied. The most commonly used method is to calculate the mean *μ* and standard deviation *σ* of grey-levels within the ROI/VOI and to exclude grey-levels outside the range *μ ± 3σ* [17–19].

**Fig. 2:**
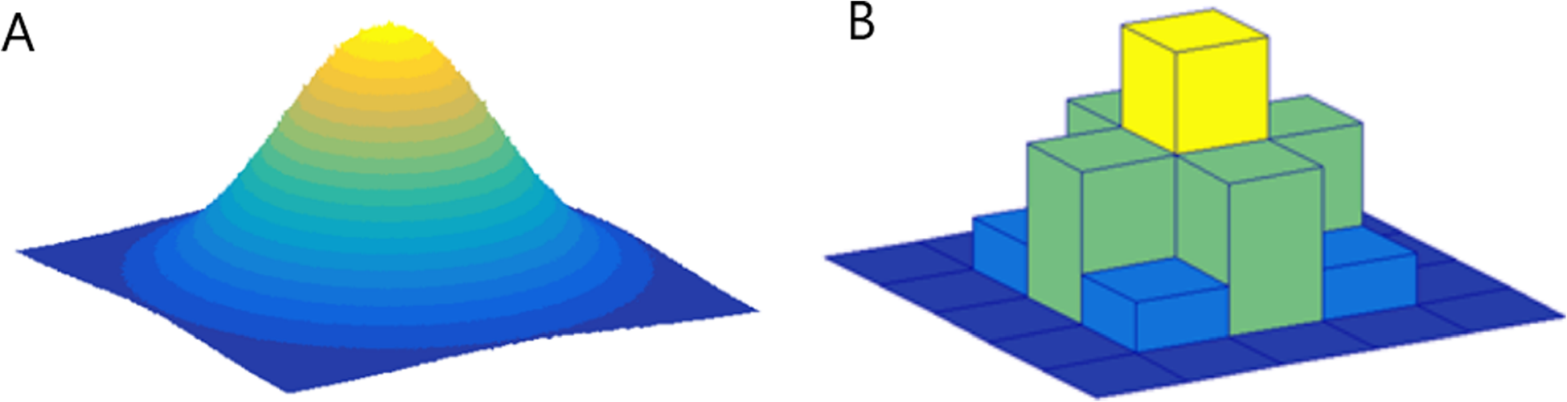
Image intensity discretization. Original data (**a**) and a generic discretized version (**b**)

The last image processing step is *discretization* of image intensities inside the ROI/VOI (Fig. 2). Discretization consists in grouping the original values according to specific range intervals (bins); the procedure is conceptually equivalent to the creation of a histogram. This step is required to make feature calculation tractable [20].

Three parameters characterize discretization: the range of the discretized quantity, the number of bins, and their width (size). The range equals the product of the bin number times the bin width; therefore, only two of the parameters can be freely set. Different combinations can lead to different results; the choice of the three parameters is usually influenced by the context, e.g., to simplify the comparison with other works using a particular binning:

- The range is usually preserved from the original data, but exceptions are not uncommon, e.g. when the discretized data is to be compared with some reference dataset or when ROIs with much smaller range than the original have to be analyzed. It is worth mentioning that when the range is not preserved and if the number of bins is particularly small, the choice of the range boundaries can have a strong impact on the results;
- Fixing the bin number (as is the case of discretizing grey-level intensities) normalizes images and is especially beneficial in data with arbitrary intensity units (e.g., MRI) and where contrasts are considered important [17]. Thus, it is the recommended discretization method for MRI data, although this recommendation is not without controversies (for further discussion, please refer to the relative pyRadiomics documentation^9^). The use of a fixed bin number discretization is thought to make radiomic features more reproducible across different samples, since the absolute values of many features depend on the number of grey levels within the ROI/VOI;
- Fixing the bin size results in having direct control on the absolute range represented on each bin, therefore allowing the bin sequence to have an immediate relationship with the original intensity scale (such as Hounsfield units or standardized uptake values). This approach makes it possible to compare discretized data with different ranges, since the bins belonging to the overlapping range will represent the same data interval. For that reason, previous work recommends the use of a fixed bin size for PET images [14]. It is recommended to use identical minimum values for all samples, defined by the lower bound of the re-segmentation range

A still open question is the optimal bin number/bin width which should be used in this discretization step. This question becomes particularly important when considering that the discretization is equivalent to averaging the values within each bin, and the effect is similar to applying a smoothing filter on the data distribution. When the bins are too wide (too few), features can be averaged out and lost; when the bins are too small (too many), features can become indistinguishable from noise. A balance is reached when discretization can filter out the noise while preserving the interesting features; unfortunately, this implies that the optimal choice of binning is highly dependent from the both data acquisition parameters (noise) and content (features). As an example, previous preliminary work has shown that different MRI sequences might need different bin numbers for obtaining robust and reproducible radiomics features [11]. Moreover, small number of bins can generate undesired dependencies on the particular choice of range and bin boundaries, thus undermining the robustness of the analysis. The present recommendation is to always start by inspecting the histogram of the data from which radiomic features are to be extracted and to decide upon a reasonable set of parameters for the discretization step based on the experience.

### Step 3: feature extraction

After image segmentation and processing, extraction of radiomic features can finally be performed. Feature extraction refers to the calculation of features as a final processing step, where feature descriptors are used to quantify characteristics of the grey levels within the ROI/VOI [17]. Since many different ways and formulas exist to calculate those features, adherence to the Image Biomarker Standardization Initiative (IBSI) guidelines [17] is recommended. These guidelines offer a consensus for standardized feature calculations from all radiomic feature matrices. Different types (i.e., matrices) of radiomic features exist, the most often encountered ones being intensity (histogram)-based features, shape features, texture features, transform-based features, and radial features. In addition, different types of filters (e.g., wavelet or Gaussian filters) are often applied during the feature extraction step. In practice, feature extraction means simply pressing the “run” button and waiting for the computation to be finished.

### Step 4: feature selection/dimension reduction

Depending on the software package used for feature extraction and the number of filters applied during the process, the number of extracted features to deal with during the following step of statistical analysis and machine learning ranges between a few and, in theory, unlimited. The higher the number of features/variables in a model and/or the lower the number of cases in the groups, e.g., for a classification task, the higher the risk of model overfitting.

As a consequence, reducing the number of features to build statistical and machine learning models during a step called feature selection or dimension reduction is of crucial importance for generating valid and generalizable results. Several “rules of thumb” may exist for defining the optimal number of features for a given sample size, but no true evidence for these rules exists in the literature. For some guidance regarding study design or sample size calculation, please consider reference [21]. The dimension reduction is a multi-step process, leading to exclusion of non-reproducible, redundant, and non-relevant features from the dataset.

**Fig. 3:**
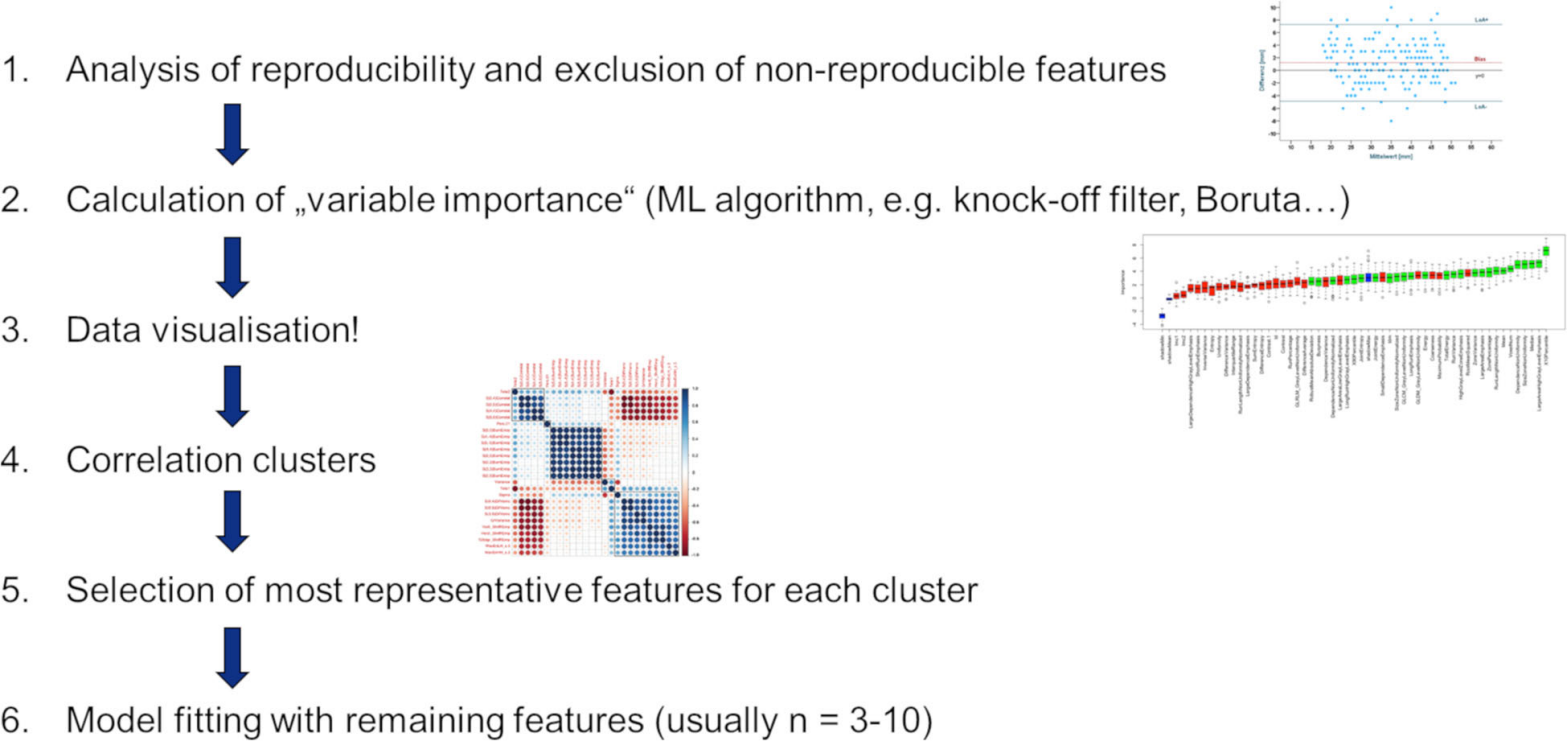
Dimension reduction and feature selection workflow

Multiple ways for dimension reduction and feature selection exist among researchers. The following steps reflect our personal experience and have been performed in several clinical studies so far [2, 22–27] (Fig. 3).

The first step should involve exclusion of non-reproducible features, if manual or semi-automated ROI/VOI delineation was used during the image segmentation step. A feature which suffers from higher intra- or interobserver variability is not likely to be informative, e.g., for assessing therapeutic response. Similarly, the test-retest robustness of the extracted features should be assessed (e.g., using a phantom). Non-robust features should also be excluded if the study aim is the evaluation of longitudinal data, although it is important that the relevant change of features over time is incorporated into the selection procedure [28]. Simply assessing reproducibility/robustness by calculation of intra-class-correlation coefficients (ICCs) might not be sufficient since ICCs are known to depend on the natural variance of the underlying data. Recommendations for assessing reproducibility, repeatability, and robustness can be found in [29].

The second step in the feature selection process is the selection of the most relevant variables for the respective task. Various approaches often relying on machine learning techniques can be used for this initial feature selection step, such as knock-off filters, recursive feature elimination methods, or random forest algorithms.

Since these algorithms often do not account for collinearities and correlations in the data, building correlation clusters represents the logical next—third—step in the dimension reduction workflow. In some cases, this step might be combined with the previous (second) step since few machine learning techniques are able to account for correlations within the data. The majority, however, is not. Correlation clusters (for an example, see Fig. 3) visualize clusters of highly correlated features in the data and allow selection of only one representative feature per correlation cluster. This selection process again might be based on machine learning algorithms and/or on conventional statistical methods and data visualization. As a general principle, the variable with the highest biological-clinical variability in the dataset should be selected since it might be most representative of the variations within the specific patient cohort. The data visualization step is also of high importance once the dimensionality of the data has been reduced.

Finally, the remaining, non-correlated and highly relevant features can be used to train the model for the respective classification task. Although the present review does not aim to cover the model training and selection process, the importance of splitting the dataset into a training and at least an independent testing dataset (for optimal conditions even an additional validation dataset) cannot be stressed enough [30]. This is especially relevant given the limitations currently encountered in the field of radiomics as discussed in the following section.

## Current limitations in radiomics

Although radiomics has shown its potential for diagnostic, prognostic, and predictive purposes in numerous studies, the field is facing several challenges. The existing gap between knowledge and clinical needs results in studies lacking clinical utility. In case a clinically relevant question is considered, the reproducibility of radiomic studies is often poor, due to lack of standardization, insufficient reporting, or limited open source code and data. Also, the lack of proper validation and the subsequent risk of false-positive results hampers the translation to clinical practice [31]. Moreover, the interpretability of the features, especially those derived from texture matrices and/or after filtering, mistakes in the interpretation of the results (e.g., causation vs. correlation), or the lack of comparison with well-established prognostic and predictive factors, results in reservation towards its use in clinical decision support systems. Furthermore, radiomics studies are often based on retrospectively collected data and thus have low level of evidence and mainly serve as proof-of-concept, whereas prospective studies are required to confirm the value of radiomics.

Due to the retrospective nature of radiomic studies, imaging protocols, including acquisition, and reconstruction settings, are often not controlled or standardized. For each image modality, multiple studies have assessed the impact of these settings on radiomic features or attempted to minimize their influence by eliminating features that are sensitive to these variabilities. Although these studies are relevant to create awareness of the influencing factors, it should be noted that the information is often not directly helpful to future studies. The reproducibility of radiomic features is not necessarily generalizable to different disease sites, modalities, or scanners, e.g., robust features in one disease site are not necessarily robust in another disease site [32]. Moreover, in case robust radiomic features are assessed using cut-off values of correlation coefficients, one should be aware that these cut-offs are often arbitrarily chosen and the number of “robust” features depend on the number of subjects involved. Furthermore, for the generalizability of robustness studies, it is important that radiomic feature calculations are compliant with the IBSI guidelines [17].

Apart from the variations in scanners and settings, radiomic feature values are also influenced by patient variabilities, e.g., geometry, which impact the levels of noise and presence of artifacts in an image. Therefore, the aim of a recent study was to quantify these so-called “non-reducible technical variations” and stabilize the radiomic features accordingly [33].

**Fig. 4:**
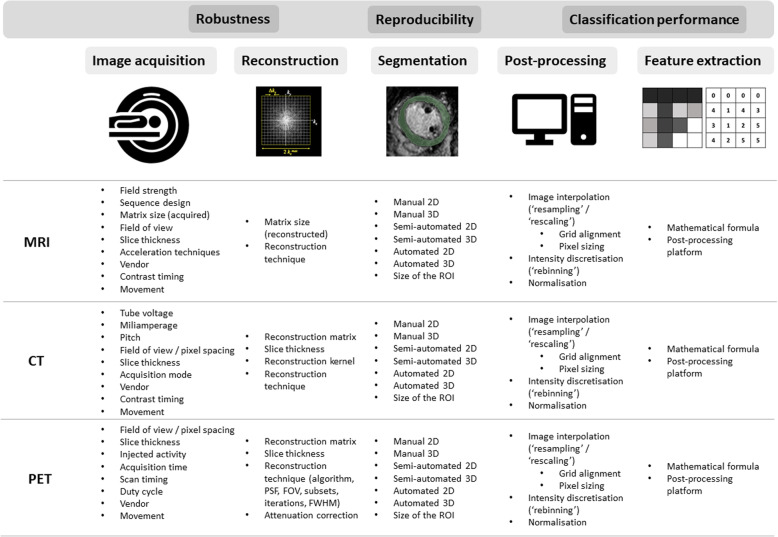
Factors influencing radiomics stability. Summary of technical factors in each step of the radiomics workflow potentially decreasing radiomic feature robustness, reproducibility, and classification performance

The next sections summarize the studies that assessed radiomic feature robustness for different acquisition and reconstruction settings of CT, PET, and MRI, as well as for ROI delineation and image pre-processing steps. Figure 4 provides an overview of factors that have been investigated in literature for their influence on radiomic feature values. In Tables 1, 2, and 3, the studies are collected in one overview for all three modalities considered in this review: CT, MRI, and PET, respectively. A recent review provides an overview of existing phantoms that have been used for radiomics for all three modalities [120].

**Table 1:** Literature review for oncologic imaging or phantom studies with computed tomography

	Ref.	Study (first author)	Year	Factor	Site/Organ
Test-retest	[34]	Du et al.	2019		NSCLC
	[35]	Mahon et al.	2019		NSCLC
	[36]	Tanaka et al.	2019		Lung cancer
	[37]	Tunali et al.	2019		NSCLC
	[38]	Zwanenburg et al.	2019		NSCLC, HNSCC
	[39]	Berenguer et al.	2018		Phantom
	[40]	Desseroit et al.	2017		NSCLC
	[41]	Larue et al.	2017		Phantom
	[42]	Larue et al.	2017		NSCLC, esophageal cancer
	[43]	Hu et al.	2016		Rectal cancer
	[32]	van Timmeren et al.	2016		NSCLC, rectal cancer
	[44]	Aerts et al.	2014		NSCLC
	[45]	Balagurunathan et al.	2014		NSCLC
	[46]	Balagurunathan et al.	2014		NSCLC
	[47]	Fried et al.	2014		NSCLC
	[48]	Hunter et al.	2013		NSCLC
Acquisition	[49]	Hepp et al.	2020	Dose	NSCLC
	[50]	Piazzese et al.	2019	Contrast	Oesophageal cancer
	[51]	Robins et al.	2019	Dose	Simulated lesions
	[36]	Tanaka et al.	2019	Breathing	Lung cancer
	[39]	Berenguer et al.	2018	Scanner, kVp, mAs, pitch, FOV, acq. mode	Phantom
	[52]	Ger et al.	2018	Scanner	Phantom
	[53]	Mackin et al.	2018	mAs	Phantom
	[54]	Shafiq-ul-Hassan et al.	2018	Scanner	Phantom
	[55]	Buch et al.	2017	kVp, mAs, pitch, acq. mode	Phantom
	[41]	Larue et al.	2017	Scanner, mAs	Phantom
	[42]	Larue et al.	2017	Breathing	NSCLC, esophageal cancer
	[56]	Mackin et al.	2017	Scanner	Phantom
	[57]	Shafiq-ul-Hassan et al.	2017	mAs, pitch	Phantom
	[58]	Lo et al.	2016	mAs	Phantom, lung nodules
	[59]	Solomon et al.	2016	Dose	Liver, lung nodules, renal stones
	[60]	Fave et al.	2015	kVp, mAs, Breathing	NSCLC
	[61]	Oliver et al.	2015	Breathing	Lung cancer
	[48]	Hunter et al.	2013	Breathing	NSCLC
Reconstruction	[62]	Choe et al.	2019	Kernel	Pulmonary nodules
	[50]	Piazzese et al.	2019	2D/3D	Oesophageal cancer
	[63]	Ligero et al.	2019	Kernel	Different tumor sites
	[51]	Robins et al.	2019	Voxel size, kernel	Simulated lesions
	[64]	Varghese et al.	2019	Voxel size, filtering	Phantom
	[39]	Berenguer et al.	2018	Voxel size, kernel	Phantom
	[54]	Shafiq-ul-Hassan et al.	2018	Voxel size	Phantom
	[55]	Buch et al.	2017	Voxel size	Phantom
	[41]	Larue et al.	2017	Voxel size	Phantom
	[56]	Mackin et al.	2017	Voxel size	Phantom
	[57]	Shafiq-ul-Hassan et al.	2017	Kernel	Phantom
	[65]	Bogowicz et al.	2016	Voxel size, calculation factors*	NSCLC, oropharyngeal carcinoma
	[66]	Kim et al.	2016	Algorithm	Pulmonary tumors
	[58]	Lo et al.	2016	Kernel	Phantom, lung nodules
	[67]	Lu et al.	2016	Algorithm, voxel size	Lung cancer
	[59]	Solomon et al.	2016	Algorithm	Liver, lung nodules, renal stones
	[68]	Zhao et al.	2016	Algorithm, voxel size	Lung cancer
	[60]	Fave et al.	2015	2D/3D	NSCLC
	[69]	Kim et al.	2015	Algorithm	Phantom
	[70]	Zhao et al.	2014	Voxel size, kernel	Phantom
Segmentation	[62]	Choe et al.	2019		Pulmonary nodules
	[63]	Ligero et al.	2019		Different tumor sites
	[71]	Qiu et al.	2019		Hepatocellular carcinoma
	[37]	Tunali et al.	2019		NSCLC
	[72]	Pavic et al.	2018		Mesothelioma, NSCLC, HN
	[73]	Kalpathy-Cramer et al.	2016		Lung nodules, phantom
	[44]	Aerts et al.	2014		NSCLC
	[45]	Balagurunathan et al.	2014		NSCLC
	[74]	Parmar et al.	2014		Lung cancer
Image processing	[75]	Lee et al.	2019	Discretization, resampling	Lung cancer
	[52]	Ger et al.	2018	Discretization, HU threshold, filtering	Phantom
	[57]	Shafiq-ul-Hassan et al.	2017	Resampling	Phantom
	[76]	Bagher-Ebadian et al.	2017	Filtering	Oropharyngeal cancer
	[41]	Larue et al.	2017	Discretization	Phantom
	[56]	Mackin et al.	2017	Resampling, filtering	Phantom
	[65]	Bogowicz et al.	2016	Discretization*	NSCLC, Oropharyngeal carcinoma
	[60]	Fave et al.	2016	Resampling, filtering	NSCLC

*In this study, CT perfusion maps were in vestigated

**Table 2:** Literature review for oncologic imaging or phantom studies with positron emission tomography

	Ref.	Study (first author)	Year	Factor	Site/Organ
Test-retest	[77]	Konert et al.	2020		NSCLC
	[78]	Vuong et al.	2019		Lung cancer
	[79]	Gallivanone et al.	2018		Phantom
	[40]	Desseroit et al.	2017		NSCLC
	[80]	Leijenaar et al.	2013		NSCLC
Acquisition	[77]	Konert et al.	2020	Breathing	NSCLC
	[81]	Pfaehler et al.	2019	Acquisition time	Phantom
	[82]	Branchini et al.	2019	Injected activity	Pedriatic cancer
	[78]	Vuong et al.	2019	Breathing	Lung cancer
	[83]	Charles et al.	2017	Breathing	Phantom
	[84]	Lovat et al.	2017	Scan timing	Neurofibromatosis-1
	[85]	Reuzé et al.	2017	Scanner	Cervical cancer
	[86]	Shiri et al.	2017	Acquisition time	Phantom, lung, HN, liver cancer
	[13]	Bailly et al.	2016	Acquisition time	Neuroendocrine tumors
	[87]	Forgacs et al.	2016	Acquisition time	Phantom, lung cancer
	[88]	Grootjans et al.	2016	Breathing, duty cycle	Lung cancer
	[89]	Nyflot et al.	2015	Injected activity, acquisiton time	Simulated phantom
Reconstruction	[81]	Pfaehler et al.	2019	Algorithm, PSF, FWHM	Phantom
	[79]	Gallivanone et al.	2018	PSF, TOF, matrix size, iterations, subsets, FWHM	Phantom
	[12]	Altazi et al.	2017	Algorithm	Cervical tumor
	[86]	Shiri et al.	2017	PSF, TOF, iterations, subsets, FWHM, matrix size	Phantom, lung, HN, liver cancer
	[13]	Bailly et al.	2016	Algorithm, iterations, FWHM, matrix size	Neuroendocrine tumors
	[90]	Cheng et al.	2016	Attenuation correction	NSCLC
	[87]	Forgacs et al.	2016	Algorithm, TOF, FWHM, voxel size	Phantom, lung cancer
	[91]	Lasnon et al.	2016	PSF, FWHM	Lung cancer
	[92]	van Velden et al.	2016	Algorithm	NSCLC
	[93]	Doumou et al.	2015	FWHM	Esophageal cancer
	[89]	Nyflot et al.	2015	Iterations, FWHM	Phantom
	[94]	Yan et al.	2015	PSF, TOF, iterations, FWHM, matrix size	Lung cancer
Segmentation	[77]	Konert et al.	2020		NSCLC
	[95]	Yang et al.	2020		Simulated lung lesions
	[81]	Pfaehler et al.	2019		Phantom
	[78]	Vuong et al.	2019		Lung cancer
	[79]	Gallivanone et al.	2018		Phantom
	[96]	Hatt et al.	2018		NSCLC, HN, simulated lesions
	[12]	Altazi et al.	2017		Cervical tumor
	[83]	Charles et al.	2017		Phantom
	[97]	Lu et al.	2016		Nasopharyngeal carcinoma
	[92]	van Velden et al.	2016		NSCLC
	[93]	Doumou et al.	2015		Esophageal cancer
	[98]	Hatt et al.	2013		Esophageal cancer
	[80]	Leijenaar et al.	2013		NSCLC
Image processing	[77]	Konert et al.	2020	Discretization	NSCLC
	[95]	Yang et al.	2020	Discretization	Simulated lung lesions
	[82]	Branchini et al.	2019	Discretization	Pedriatic cancer
	[87]	Forgacs et al.	2019	Discretization	Lung cancer
	[81]	Pfaehler et al.	2019	Discretization	Phantom
	[99]	Whybra et al.	2019	Resampling	Esophageal cancer
	[100]	Presotto et al.	2018	Discretization	Phantom
	[12]	Altazi et al.	2017	Discretization	Cervical cancer
	[85]	Reuzé et al.	2017	Resampling	Cervical cancer
	[101]	Yip et al.	2017	Discretization, resampling	NSCLC
	[97]	Lu et al.	2016	Discretization	Nasopharyngeal carcinoma
	[92]	van Velden et al.	2016	Discretization	NSCLC
	[93]	Doumou et al.	2015	Discretization	Esophageal cancer
	[14]	Leijenaar et al.	2015	Discretization	NSCLC

**Table 3:** Literature review for oncologic imaging or phantom studies with magnetic resonance imaging

	Ref.	Study (first author)	Year	Factor	Site/Organ
Test-retest	[102]	Bianchini et al.	2020		Phantom
	[9]	Baessler et al.	2019		Phantom
	[103]	Fiset et al.	2019		Cervical cancer
	[35]	Mahon et al.	2019		NSCLC
	[104]	Peerlings et al.	2019		Ovarian cancer, lung cancer, colorectal liver metastasis
	[105]	Schwier et al.	2019		Prostate
Acquisition	[9]	Baessler et al.	2019	Matrix size	Phantom
	[106]	Bologna et al.	2019	TR, TE, INU, noise level	Phantom
	[107]	Cattell et al.	2019	Noise level	Phantom
	[103]	Fiset et al.	2019	Scanner	Cervical cancer
	[108]	Um et al.	2019	Scanner, field strength	Glioblastoma
	[109]	Yang et al.	2018	Noise level, accelerator factor	Phantom, glioma
Reconstruction	[9]	Baessler et al.	2019	Matrix size	Phantom
	[106]	Bologna et al.	2019	Voxel size	Phantom
	[107]	Cattell et al.	2019	Voxel size	Phantom
	[109]	Yang et al.	2018	Algorithm	Phantom, glioma
Segmentation	[110]	Traverso et al.	2020		Cervical cancer
	[9]	Baessler et al.	2019		Phantom
	[107]	Cattell et al.	2019		Phantom
	[111]	Duron et al.	2019		Lacrymal gland tumors, breast lesions
	[103]	Fiset et al.	2019		Cervical cancer
	[112]	Tixier et al.	2019		Glioblastoma
	[113]	Zhang et al.	2019		Nasopharyngeal carcinoma, sentinel lymph node
	[114]	Saha et al.	2018		Breast cancer
	[115]	Veeraraghavan et al.	2018		Breast cancer
Image processing	[116]	Isaksson et al.	2020	Normalization	Prostate cancer
	[117]	Scalco et al.	2020	Normalization	Prostate cancer
	[110]	Traverso et al.	2020	Normalization, discretization, filtering	Cervical cancer
	[106]	Bologna et al.	2019	Normalization, resampling, filtering	Phantom
	[111]	Duron et al.	2019	Discretization	Lacrymal gland tumors, breast lesions
	[118]	Moradmand et al.	2019	Bias field correction, filtering	Glioblastoma
	[119]	Um et al.	2019	Bias field correction, normalization, discretization, filtering	Glioblastoma

### CT and PET CT

Multiple studies (16 were identified in this review) have investigated the stability over test-retest scenarios for CT radiomics (Table 1), where the publicly available RIDER Lung CT collection was often evaluated [121]. For PET, only a few test-retest studies were performed, which were either on a phantom or lung cancer data (Table 2). Recently, an extensive review on factors influencing PET radiomics was published [122].

The voxel size was the mostly investigated influencing reconstruction factor for CT, whereas this was the full-width half maximum (FWHM) of the Gaussian filter for PET. Four and 12 studies were identified that studied the influence of image discretization on CT and PET radiomic features, respectively. Figure 4 provides an overview of factors that have been investigated in literature for their influence on radiomic feature values.

### MRI

The impact of test-retest, acquisition and reconstruction settings, segmentation, and image pre-processing has been explored less extensively to date than for PET and CT. Only four studies were found that investigated the influence of reconstruction settings, one of these studies included patient images. The influence of segmentation on MRI radiomic features has been more extensively studied for a variety of tumor sites. Table 3 summarizes the present literature for influencing factors on radiomic features in MRI. Figure 4 provides an overview of factors that have been investigated in literature for their influence on radiomic feature values.

### Reduce radiomics’ dependency

Recent literature regarding the robustness for different acquisition and reconstruction settings, ROI delineation, and image pre-processing steps shows that the most commonly used approach to deal with this is to eliminate radiomic features that are not robust against these factors. The drawback of this method is that potentially relevant information could be removed, whereas stability not necessarily means informativity. A few solutions have been proposed in order to reduce the influence of the aforementioned factors on radiomics studies. One proposed solution is to eliminate the dependency of features on a certain factor by modeling the relationship and applying corrections accordingly. This had been explored recently for different CT exposure settings [123]. Another method to eliminate the dependency is to convert images using deep learning, in order to simulate reconstruction with different settings, which was shown to improve CT radiomics’ reproducibility for images reconstructed with different kernels [62]. This approach has the potential to solve other radiomics dependencies to improve robustness in the future. Different than image-wise dependency corrections, post-reconstruction batch harmonization has been proposed in order to harmonize radiomic feature sets originating from different institutes, which is a method called ComBat [124–126]. Furthermore, a recent study investigated the performance of data augmentation instead of feature elimination to incorporate the knowledge on influencing factors on radiomic features [127].

### Open-source data

Publicly available datasets like the RIDER dataset 10 help to gain knowledge about the impact of varying factors in radiomics [121]. Also, the availability of a public phantom dataset, intended for radiomics reproducibility tests on CT, could help to further assess the influence of acquisition settings in order to eliminate non-robust radiomic features [128]. However, studies are needed to show if robustness data acquired on a phantom can be translated to the human. Similar initiatives for PET and MRI would help to understanding of the impact of changes in settings on radiomics. In other words, open-source data plays an important role in the future improvement of radiomics.

### Solution: quality control and standardization

In order to increase the chance of clinically relevant and valuable radiomics studies, we would recommend verifying whether the following questions could be answered with “yes,” prior to commencement of the study:

- Is there an actual clinical need which could potentially be answered with (the help of) radiomics?
- Is there enough expertise in the research team, preferably from at least two different disciplines, to ensure high quality of the study and potential of clinical implementation?
- Is there access to enough data to support the conclusions with sufficient power, including external validation datasets?
- Is it possible to retrieve all other non-imaging data that is known to be relevant for the research question (e.g., from biological information, demographics)?
- Is information on the acquisition and reconstruction of the images available?
- Are the imaging protocols standardized and if not, is there a solution to harmonize images or to ensure minimal influence of varying settings on the modeling?

Besides these general questions, which should been asked before the start of a study, there are some recent contributions in the field that aim to facilitate the execution of radiomics studies with higher quality: (1) IBSI: harmonization of radiomics implementations and guidelines on reporting of radiomic studies [17, 129], (2) Radiomics Quality Score (RQS): checklist to ensure quality of radiomics studies [130], and (3) *Transparent reporting of a multivariable prediction model for individual prognosis or diagnosis* (TRIPOD) statement—guidelines for reporting of prediction models for prognosis or diagnosis [30]. For the radiomic feature calculation, we recommend to use an implementation that is IBSI compliant, which could be verified using the publicly available digital phantom [129, 130]. Also, regarding choices for image discretization and resampling, we recommend following the IBSI guidelines. Besides that, it is important to be consistent and transparent, and detailed reporting on the pre-processing steps applied to improve reproducibility and repeatability of radiomic studies need to be ensured.

A recent study evaluated the quality of 77 oncology-related radiomics studies using RQS and TRIPOD, and concluded that “the overall scientific quality and reporting of radiomics studies is insufficient,” showing the importance of guidelines and criteria for future studies [131].

### Outlook: workflow integration

While currently many research efforts aim towards standardization of radiomics, translation into clinical practice also requires adequate implementation of radiomics analyses into the clinical workflow once the standardization issue has been adequately addressed and clinical utility has been proven in prospective clinical trials.

A useful radiomics tool should seamlessly integrate into the clinical radiological workflow and be incorporated into or interfaced with existing RIS/PACS systems. Such systems should provide segmentation tools or ideally deep learning-based automated segmentation methods as well as standardized feature extraction algorithms and modality-adjusted image processing adhering to the standards described above. In case of fully automated segmentation, the possibility to inspect and manually correct the segmentation results should be incorporated.

In a future workflow, known important radiomics features could then be displayed alongside other quantitative imaging biomarkers and the images themselves. The radiologist could then use all these information to support his clinical judgement or—where possible—estimate, e.g., prognostic factors.

It is, however, important to note, that radiomics should only be viewed as an additional tool and not as a standalone diagnostic algorithm. Certainly, many challenges lie ahead until radiomics can be integrated in our daily routine: from the above-mentioned issues surrounding image standardization to legal issues that will certainly arise regarding regulatory issues. Nonetheless, it could prove a valuable if not critical step towards a more integrated approach to healthcare.

## Conclusions

Throughout the radiomics workflow, multiple factors have been identified that influence the feature values, including random variations in scanner and patients, image acquisition and reconstruction settings, ROI segmentation, and image preprocessing. Several studies have proposed to either eliminate unstable features, correct for influencing factors, or harmonize datasets in order to improve the robustness of radiomics. Recently published guidelines and checklists aim to improve the quality of future radiomics studies, but transparency has been recognized as the most important factor for reproducibility. Assessment of clinical relevance and impact prior to study commencement, increased level of evidence using studies with large enough datasets and external validation, and its combination with established methods will help moving the field towards clinical implementation.

## Data Availability

Not applicable.

## Abbreviations

AI: Artificial intelligence
CT: Computed tomography
DL: Deep learning
IBSI: Imaging Biomarker Standardization Initiative
ICC: Intra-class-correlation coefficient
ML: Machine learning
MRI: Magnetic resonance imaging
PET: Positron emission tomography
ROI: Region of interest
RQS: Radiomics Quality Score
TRIPOD: Transparent reporting of a multivariable prediction model for individual prognosis or diagnosis
VOI: Volume of interest

## Footnotes

1 TechAmerica Foundation’s Federal Big Data Commission, 2012 https://bigdatawg.nist.gov/_uploadfiles/M0068_v1_3903747095.pdf

2 https://slicer.org

3 https://mitk.org

4 https://itksnap.org

5 https://mevislab.de

6 https://lifexsoft.org

7 https://imagej.nih.gov

8 https://github.com/Radiomics/pyradiomics/tree/master/examples/exampleSettings

9 https://pyradiomics.readthedocs.io/en/latest/faq.html#radiomics-fixed-bin-width

10 https://wiki.cancerimagingarchive.net/display/Public/RIDER+Lung+CT

## References

[1] Neisius U, El-Rewaidy H, Nakamori S, Rodriguez J, Manning WJ, Nezafat R (2019). Radiomic analysis of myocardial native T1 imaging discriminates between hypertensive heart disease and hypertrophic cardiomyopathy. JACC Cardiovasc Imaging.

[2] Mannil M, von Spiczak J, Manka R, Alkadhi H (2018). Texture analysis and machine learning for detecting myocardial infarction in noncontrast low-dose computed tomography: unveiling the invisible. Invest Radiol.

[3] Castellano G, Bonilha L, Li LM, Cendes F (2004). Texture analysis of medical images. Clin Radiol.

[4] Tourassi GD (1999). Journey toward computer-aided diagnosis: role of image texture analysis. Radiology.

[5] Fedorov A, Beichel R, Kalpathy-Cramer J (2012). 3D Slicer as an image computing platform for the Quantitative Imaging Network. Magn Reson Imaging.

[6] Abràmoff MD, Magalhães PJ, Ram SJ (2004). Image processing with ImageJ. Biophotonics Int.

[7] Kresanova Z, Kostolny J. Comparison of Software for Medical Segmentation, p 15

[8] Lee L-K, Liew S-C (2015). A survey of medical image processing tools.

[9] Baeßler B, Weiss K, Pinto dos Santos D (2019). Robustness and reproducibility of radiomics in magnetic resonance imaging: a phantom study. Invest Radiol.

[10] Ronneberger O, Fischer P, Brox T (2015) U-Net: convolutional networks for biomedical image segmentation. arXiv:1505.04597

[11] Wichtmann B, Attenberger U, Harder FM (2018). Influence of image processing on the robustness of radiomic features derived from magnetic resonance imaging—a phantom study. ISMRM 2018.

[12] Altazi BA, Zhang GG, Fernandez DC (2017). Reproducibility of F18-FDG PET radiomic features for different cervical tumor segmentation methods, gray-level discretization, and reconstruction algorithms. J Appl Clin Med Phys.

[13] Bailly C, Bodet-Milin C, Couespel S (2016). Revisiting the robustness of PET-based textural features in the context of multi-centric trials. PLoS One.

[14] Leijenaar RTH, Nalbantov G, Carvalho S (2015). The effect of SUV discretization in quantitative FDG-PET Radiomics: the need for standardized methodology in tumor texture analysis. Sci Rep.

[15] Shafiq-ul-Hassan M, Zhang GG, Latifi K (2017). Intrinsic dependencies of CT radiomic features on voxel size and number of gray levels. Med Phys.

[16] van Griethuysen JJM, Fedorov A, Parmar C (2017). Computational radiomics system to decode the radiographic phenotype. Cancer Res.

[17] Zwanenburg A, Leger S, Vallières M, Löck S (2016) Image biomarker standardisation initiative. arXiv:1612.07003

[18] Collewet G, Strzelecki M, Mariette F (2004). Influence of MRI acquisition protocols and image intensity normalization methods on texture classification. Magn Reson Imaging.

[19] Vallières M, Freeman CR, Skamene SR, Naqa IE (2015). A radiomics model from joint FDG-PET and MRI texture features for the prediction of lung metastases in soft-tissue sarcomas of the extremities. Phys Med Biol.

[20] Yip SSF, Aerts HJWL (2016). Applications and limitations of radiomics. Phys Med Biol.

[21] Riley RD, Snell KI, Ensor J (2019). Minimum sample size for developing a multivariable prediction model: PART II - binary and time-to-event outcomes. Stat Med.

[22] Baessler B, Mannil M, Oebel S, Maintz D, Alkadhi H, Manka R (2018). Subacute and chronic left ventricular myocardial scar: accuracy of texture analysis on nonenhanced Cine MR images. Radiology.

[23] Baessler B, Luecke C, Lurz J (2018). Cardiac MRI texture analysis of T1 and T2 maps in patients with infarctlike acute myocarditis. Radiology.

[24] Baessler B, Luecke C, Lurz J (2019). Cardiac MRI and texture analysis of myocardial T1 and T2 maps in myocarditis with acute versus chronic symptoms of heart failure. Radiology.

[25] Baeßler B, Mannil M, Maintz D, Alkadhi H, Manka R (2018). Texture analysis and machine learning of non-contrast T1-weighted MR images in patients with hypertrophic cardiomyopathy-preliminary results. Eur J Radiol.

[26] Baessler B, Nestler T, Pinto dos Santos D (2020). Radiomics allows for detection of benign and malignant histopathology in patients with metastatic testicular germ cell tumors prior to post-chemotherapy retroperitoneal lymph node dissection. Eur Radiol.

[27] Di Noto T, von Spiczak J, Mannil M (2019). Radiomics for distinguishing myocardial infarction from myocarditis at late gadolinium enhancement at MRI: comparison with subjective visual analysis. Radiol Cardiothorac Imaging.

[28] van Timmeren JE, Leijenaar RTH, van Elmpt W, Reymen B, Lambin P (2017). Feature selection methodology for longitudinal cone-beam CT radiomics. Acta Oncol.

[29] Sullivan DC, Obuchowski NA, Kessler LG (2015). Metrology standards for quantitative imaging biomarkers. Radiology.

[30] Collins GS, Reitsma JB, Altman DG, Moons KGM (2015). Transparent reporting of a multivariable prediction model for individual prognosis or diagnosis (TRIPOD): the TRIPOD statement. BMJ.

[31] Chalkidou A, O’Doherty MJ, Marsden PK (2015). False discovery rates in PET and CT studies with texture features: a systematic review. PLoS One.

[32] van Timmeren J, Leijenaar RTH, van Elmpt W (2016). Test–retest data for radiomics feature stability analysis: generalizable or study-specific?. Tomography.

[33] Mühlberg A, Katzmann A, Heinemann V (2020). The technome - a predictive internal calibration approach for quantitative imaging biomarker research. Sci Rep.

[34] Du Q, Baine M, Bavitz K (2019). Radiomic feature stability across 4D respiratory phases and its impact on lung tumor prognosis prediction. PLoS One.

[35] Mahon RN, Hugo GD, Weiss E (2019). Repeatability of texture features derived from magnetic resonance and computed tomography imaging and use in predictive models for non-small cell lung cancer outcome. Phys Med Biol.

[36] Tanaka S, Kadoya N, Kajikawa T (2019). Investigation of thoracic four-dimensional CT-based dimension reduction technique for extracting the robust radiomic features. Phys Med.

[37] Tunali I, Hall LO, Napel S (2019). Stability and reproducibility of computed tomography radiomic features extracted from peritumoral regions of lung cancer lesions. Med Phys.

[38] Zwanenburg A, Leger S, Agolli L (2019). Assessing robustness of radiomic features by image perturbation. Sci Rep.

[39] Berenguer R, Pastor-Juan MDR, Canales-Vázquez J (2018). Radiomics of CT features may be nonreproducible and redundant: influence of CT acquisition parameters. Radiology.

[40] Desseroit M-C, Tixier F, Weber WA (2017). Reliability of PET/CT shape and heterogeneity features in functional and morphologic components of non–small cell lung cancer tumors: a repeatability analysis in a prospective multicenter cohort. J Nucl Med.

[41] Larue RTHM, van Timmeren JE, de Jong EEC (2017). Influence of gray level discretization on radiomic feature stability for different CT scanners, tube currents and slice thicknesses: a comprehensive phantom study. Acta Oncol.

[42] Larue RTHM, Van De Voorde L, van Timmeren JE (2017). 4DCT imaging to assess radiomics feature stability: An investigation for thoracic cancers. Radiother Oncol.

[43] Hu P, Wang J, Zhong H et al (2016) Reproducibility with repeat CT in radiomics study for rectal cancer. Oncotarget 7 10.18632/oncotarget.12199 10.18632/oncotarget.12199 PMC5342090 27669756

[44] Aerts HJWL, Velazquez ER, Leijenaar RTH (2014). Decoding tumour phenotype by noninvasive imaging using a quantitative radiomics approach. Nat Commun.

[45] Balagurunathan Y, Gu Y, Wang H (2014). Reproducibility and prognosis of quantitative features extracted from CT images. Transl Oncol.

[46] Balagurunathan Y, Kumar V, Gu Y (2014). Test–retest reproducibility analysis of lung CT image features. J Digit Imaging.

[47] Fried DV, Tucker SL, Zhou S (2014). Prognostic value and reproducibility of pretreatment ct texture features in stage III non-small cell lung cancer. Int J Radiat Oncol.

[48] Hunter LA, Krafft S, Stingo F (2013). High quality machine-robust image features: Identification in nonsmall cell lung cancer computed tomography images: Robust quantitative image features. Med Phys.

[49] Hepp T, Othman A, Liebgott A, Kim JH, Pfannenberg C, Gatidis S (2020). Effects of simulated dose variation on contrast-enhanced CT-based radiomic analysis for Non-Small Cell Lung Cancer. Eur J Radiol.

[50] Piazzese C, Foley K, Whybra P, Hurt C, Crosby T, Spezi E (2019). Discovery of stable and prognostic CT-based radiomic features independent of contrast administration and dimensionality in oesophageal cancer. PLoS One.

[51] Robins M, Solomon J, Hoye J, Abadi E, Marin D, Samei E (2019). Systematic analysis of bias and variability of texture measurements in computed tomography. J Med Imaging.

[52] Ger RB, Zhou S, Chi P-CM (2018). Comprehensive investigation on controlling for CT imaging variabilities in radiomics studies. Sci Rep.

[53] Mackin D, Ger R, Dodge C (2018). Effect of tube current on computed tomography radiomic features. Sci Rep.

[54] Shafiq-ul-Hassan M, Latifi K, Zhang G, Ullah G, Gillies R, Moros E (2018). Voxel size and gray level normalization of CT radiomic features in lung cancer. Sci Rep.

[55] Buch K, Li B, Qureshi MM, Kuno H, Anderson SW, Sakai O (2017). Quantitative assessment of variation in CT parameters on texture features: pilot study using a nonanatomic phantom. AJNR Am J Neuroradiol.

[56] Mackin D, Fave X, Zhang L (2017). Harmonizing the pixel size in retrospective computed tomography radiomics studies. PLoS One.

[57] Shafiq-ul-Hassan M, Zhang GG, Hunt DC (2017). Accounting for reconstruction kernel-induced variability in CT radiomic features using noise power spectra. J Med Imaging.

[58] Lo P, Young S, Kim HJ, Brown MS, McNitt-Gray MF (2016). Variability in CT lung-nodule quantification: Effects of dose reduction and reconstruction methods on density and texture based features: Variability in CT lung-nodule quantification. Med Phys.

[59] Solomon J, Mileto A, Nelson RC, Choudhury KR, Samei E (2016). Quantitative features of liver lesions, lung nodules, and renal stones at multi–detector row CT examinations: dependency on radiation dose and reconstruction algorithm. Radiology.

[60] Fave X, Cook M, Frederick A (2015). Preliminary investigation into sources of uncertainty in quantitative imaging features. Comput Med Imaging Graph.

[61] Oliver JA, Budzevich M, Zhang GG, Dilling TJ, Latifi K, Moros EG (2015). Variability of image features computed from conventional and respiratory-gated PET/CT images of lung cancer. Transl Oncol.

[62] Choe J, Lee SM, Do K-H (2019). Deep learning–based image conversion of CT reconstruction kernels improves radiomics reproducibility for pulmonary nodules or masses. Radiology.

[63] Ligero M, Torres G, Sanchez C, Diaz-Chito K, Perez R, Gil D (2019). Selection of radiomics features based on their reproducibility. 2019 41st Annual International Conference of the IEEE Engineering in Medicine and Biology Society (EMBC).

[64] Varghese BA, Hwang D, Cen SY (2019). Reliability of CT-based texture features: Phantom study. J Appl Clin Med Phys.

[65] Bogowicz M, Riesterer O, Bundschuh RA (2016). Stability of radiomic features in CT perfusion maps. Phys Med Biol.

[66] Kim H, Park CM, Lee M (2016). Impact of reconstruction algorithms on CT radiomic features of pulmonary tumors: analysis of intra- and inter-reader variability and inter-reconstruction algorithm variability. PLoS One.

[67] Lu L, Ehmke RC, Schwartz LH, Zhao B (2016). Assessing agreement between radiomic features computed for multiple CT imaging settings. PLoS One.

[68] Zhao B, Tan Y, Tsai W-Y (2016). Reproducibility of radiomics for deciphering tumor phenotype with imaging. Sci Rep.

[69] Kim HG, Chung YE, Lee YH (2015). Quantitative analysis of the effect of iterative reconstruction using a phantom: determining the appropriate blending percentage. Yonsei Med J.

[70] Zhao B, Tan Y, Tsai WY, Schwartz LH, Lu L (2014). Exploring Variability in CT characterization of tumors: a preliminary phantom study. Transl Oncol.

[71] Qiu Q, Duan J, Duan Z (2019). Reproducibility and non-redundancy of radiomic features extracted from arterial phase CT scans in hepatocellular carcinoma patients: impact of tumor segmentation variability. Quant Imaging Med Surg.

[72] Pavic M, Bogowicz M, Würms X (2018). Influence of inter-observer delineation variability on radiomics stability in different tumor sites. Acta Oncol.

[73] Kalpathy-Cramer J, Mamomov A, Zhao B (2016). Radiomics of lung nodules: a multi-institutional study of robustness and agreement of quantitative imaging features. Tomography.

[74] Parmar C, Rios Velazquez E, Leijenaar R (2014). Robust radiomics feature quantification using semiautomatic volumetric segmentation. PLoS ONE.

[75] Lee S-H, Cho H, Lee HY, Park H (2019). Clinical impact of variability on CT radiomics and suggestions for suitable feature selection: a focus on lung cancer. Cancer Imaging.

[76] Bagher‐Ebadian H, Siddiqui F, Liu C, Movsas B, Chetty IJ (2017). On the impact of smoothing and noise on robustness of CT and CBCT radiomics features for patients with head and neck cancers. Med Phys.

[77] Konert T, Everitt S, La Fontaine MD (2020). Robust, independent and relevant prognostic 18F-fluorodeoxyglucose positron emission tomography radiomics features in non-small cell lung cancer: Are there any?. PLoS One.

[78] Vuong D, Tanadini-Lang S, Huellner MW (2019). Interchangeability of radiomic features between [18F]- FDG PET / CT and [18F]- FDG PET / MR. Med Phys.

[79] Gallivanone F, Interlenghi M, D’Ambrosio D, Trifirò G, Castiglioni I (2018). Parameters influencing PET imaging features: a phantom study with irregular and heterogeneous synthetic lesions. Contrast Media Mol Imaging.

[80] Leijenaar RTH, Carvalho S, Velazquez ER (2013). Stability of FDG-PET Radiomics features: An integrated analysis of test-retest and inter-observer variability. Acta Oncol.

[81] Pfaehler E, Beukinga RJ, de Jong JR (2019). Repeatability of ^18^ F-FDG PET radiomic features: A phantom study to explore sensitivity to image reconstruction settings, noise, and delineation method. Med Phys.

[82] Branchini M, Zorz A, Zucchetta P (2019). Impact of acquisition count statistics reduction and SUV discretization on PET radiomic features in pediatric 18F-FDG-PET/MRI examinations. Phys Med.

[83] Carles M, Torres-Espallardo I, Alberich-Bayarri A (2017). Evaluation of PET texture features with heterogeneous phantoms: complementarity and effect of motion and segmentation method. Phys Med Biol..

[84] Lovat E, Siddique M, Goh V, Ferner RE, Cook GJ, Warbey VS (2017). The effect of post-injection 18F-FDG PET scanning time on texture analysis of peripheral nerve sheath tumours in neurofibromatosis-1. EJNMMI Res.

[85] Reuzé S, Orlhac F, Chargari C et al (2017) Prediction of cervical cancer recurrence using textural features extracted from ^18^F-FDG PET images acquired with different scanners. Oncotarget 8 10.18632/oncotarget.17856 10.18632/oncotarget.17856 PMC5522136 28574816

[86] Shiri I, Rahmim A, Ghaffarian P, Geramifar P, Abdollahi H, Bitarafan-Rajabi A (2017). The impact of image reconstruction settings on 18F-FDG PET radiomic features: multi-scanner phantom and patient studies. Eur Radiol.

[87] Forgacs A, Pall Jonsson H, Dahlbom M (2016). A study on the basic criteria for selecting heterogeneity parameters of F18-FDG PET images. PLoS One.

[88] Grootjans W, Tixier F, van der Vos CS (2016). The impact of optimal respiratory gating and image noise on evaluation of intratumor heterogeneity on 18F-FDG PET imaging of lung cancer. J Nucl Med.

[89] Nyflot MJ, Yang F, Byrd D, Bowen SR, Sandison GA, Kinahan PE (2015). Quantitative radiomics: impact of stochastic effects on textural feature analysis implies the need for standards. J Med Imaging.

[90] Cheng NM, Fang YH, Tsan DL, Hsu CH, Yen TC (2016). Respiration-averaged CT for attenuation correction of PET images – impact on pet texture features in non-small cell lung cancer patients. PLoS One.

[91] Lasnon C, Majdoub M, Lavigne B (2016). 18F-FDG PET/CT heterogeneity quantification through textural features in the era of harmonisation programs: a focus on lung cancer. Eur J Nucl Med Mol Imaging.

[92] van Velden FHP, Kramer GM, Frings V (2016). Repeatability of radiomic features in non-small-cell lung cancer [18F]FDG-PET/CT studies: impact of reconstruction and delineation. Mol Imaging Biol.

[93] Doumou G, Siddique M, Tsoumpas C, Goh V, Cook GJ (2015). The precision of textural analysis in 18F-FDG-PET scans of oesophageal cancer. Eur Radiol.

[94] Yan J, Chu-Shern JL, Loi HY (2015). Impact of image reconstruction settings on texture features in 18F-FDG PET. J Nucl Med.

[95] Yang F, Simpson G, Young L, Ford J, Dogan N, Wang L (2020). Impact of contouring variability on oncological PET radiomics features in the lung. Sci Rep.

[96] Hatt M, Laurent B, Fayad H, Jaouen V, Visvikis D, Le Rest CC (2018). Tumour functional sphericity from PET images: prognostic value in NSCLC and impact of delineation method. Eur J Nucl Med Mol Imaging.

[97] Lu L, Lv W, Jiang J (2016). Robustness of Radiomic Features in [11C]Choline and [18F]FDG PET/CT Imaging of Nasopharyngeal Carcinoma: Impact of Segmentation and Discretization. Mol Imaging Biol.

[98] Hatt M, Tixier F, Le Rest CC, Pradier O, Visvikis D (2013). Robustness of intratumour 18F-FDG PET uptake heterogeneity quantification for therapy response prediction in oesophageal carcinoma. Eur J Nucl Med Mol Imaging.

[99] Whybra P, Parkinson C, Foley K, Staffurth J, Spezi E (2019). Assessing radiomic feature robustness to interpolation in 18F-FDG PET imaging. Sci Rep.

[100] Presotto L, Bettinardi V, De Bernardi E (2018). PET textural features stability and pattern discrimination power for radiomics analysis: An “ad-hoc” phantoms study. Phys Med.

[101] Yip SS, Parmar C, Kim J, Huynh E, Mak RH, Aerts HJ (2017). Impact of experimental design on PET radiomics in predicting somatic mutation status. Eur J Radiol.

[102] Bianchini L, Botta F, Origgi D (2020). PETER PHAN: An MRI phantom for the optimisation of radiomic studies of the female pelvis. Phys Med.

[103] Fiset S, Welch ML, Weiss J (2019). Repeatability and reproducibility of MRI-based radiomic features in cervical cancer. Radiother Oncol.

[104] Peerlings J, Woodruff HC, Winfield JM (2019). Stability of radiomics features in apparent diffusion coefficient maps from a multi-centre test-retest trial. Sci Rep.

[105] Schwier M, van Griethuysen J, Vangel MG (2019). Repeatability of Multiparametric Prostate MRI Radiomics Features. Sci Rep.

[106] Bologna M, Corino V, Mainardi L (2019). Technical Note: Virtual phantom analyses for preprocessing evaluation and detection of a robust feature set for MRI-radiomics of the brain. Med Phys.

[107] Cattell R, Chen S, Huang C (2019). Robustness of radiomic features in magnetic resonance imaging: review and a phantom study. Vis Comput Ind Biomed Art.

[108] Um H, Tixier F, Bermudez D, Deasy JO, Young RJ, Veeraraghavan H (2019). Impact of image preprocessing on the scanner dependence of multi-parametric MRI radiomic features and covariate shift in multi-institutional glioblastoma datasets. Phys Med Biol.

[109] Yang F, Dogan N, Stoyanova R, Ford JC (2018). Evaluation of radiomic texture feature error due to MRI acquisition and reconstruction: A simulation study utilizing ground truth. Phys Med.

[110] Traverso A, Kazmierski M, Zhovannik I (2020). Machine learning helps identifying volume-confounding effects in radiomics. Phys Med.

[111] Duron L, Balvay D, Vande Perre S (2019). Gray-level discretization impacts reproducible MRI radiomics texture features. PLoS One.

[112] Tixier F, Um H, Young RJ, Veeraraghavan H (2019) Reliability of tumor segmentation in glioblastoma: Impact on the robustness of MRI-radiomic features. Med Phys:mp.13624 10.1002/mp.13624 10.1002/mp.13624 PMC6692188 31131906

[113] Zhang X, Zhong L, Zhang B (2019). The effects of volume of interest delineation on MRI-based radiomics analysis: evaluation with two disease groups. Cancer Imaging.

[114] Saha A, Harowicz MR, Mazurowski MA (2018). Breast cancer MRI radiomics: An overview of algorithmic features and impact of inter-reader variability in annotating tumors. Med Phys.

[115] Veeraraghavan H, Dashevsky BZ, Onishi N (2018). Appearance constrained semi-automatic segmentation from DCE-MRI is reproducible and feasible for breast cancer radiomics: a feasibility study. Sci Rep.

[116] Isaksson LJ, Raimondi S, Botta F (2020). Effects of MRI image normalization techniques in prostate cancer radiomics. Phys Med.

[117] Scalco E, Belfatto A, Mastropietro A et al (2020) T2w-MRI signal normalization affects radiomics features reproducibility. Med Phys:14038 10.1002/mp.14038 10.1002/mp.14038 31971614

[118] Moradmand H, Aghamiri SMR, Ghaderi R (2020). Impact of image preprocessing methods on reproducibility of radiomic features in multimodal magnetic resonance imaging in glioblastoma. J Appl Clin Med Phys.

[119] Um H, Tixier F, Bermudez D, Deasy JO, Young RJ, Veeraraghavan H (2019). Impact of image preprocessing on the scanner dependence of multi-parametric MRI radiomic features and covariate shift in multi-institutional glioblastoma datasets. Phys Med Biol.

[120] Valladares A, Beyer T, Rausch I (2020) Physical imaging phantoms for simulation of tumor heterogeneity in PET, CT, and MRI: An overview of existing designs. Med Phys:mp.14045 10.1002/mp.14045 10.1002/mp.14045 PMC7216968 31981214

[121] Zhao B, James LP, Moskowitz CS (2009). Evaluating variability in tumor measurements from same-day repeat CT scans of patients with non–small cell lung cancer. Radiology.

[122] Zwanenburg A (2019). Radiomics in nuclear medicine: robustness, reproducibility, standardization, and how to avoid data analysis traps and replication crisis. Eur J Nucl Med Mol Imaging.

[123] Zhovannik I, Bussink J, Traverso A (2019). Learning from scanners: bias reduction and feature correction in radiomics. Clin Transl Radiat Oncol.

[124] Orlhac F, Boughdad S, Philippe C (2018). A postreconstruction harmonization method for multicenter radiomic studies in PET. J Nucl Med.

[125] Orlhac F, Frouin F, Nioche C, Ayache N, Buvat I (2019). Validation of A Method to Compensate Multicenter Effects Affecting CT Radiomics. Radiology.

[126] Mahon RN, Ghita M, Hugo GD, Weiss E (2020). ComBat harmonization for radiomic features in independent phantom and lung cancer patient computed tomography datasets. Phys Med Biol.

[127] Götz M, Maier-Hein KH (2020). Optimal statistical incorporation of independent feature stability information into radiomics studies. Sci Rep.

[128] Kalendralis P, Traverso A, Shi Z (2019). Multicenter CT phantoms public dataset for radiomics reproducibility tests. Med Phys.

[129] Zwanenburg A, Vallières M, Abdalah MA et al (2020) The image biomarker standardization initiative: standardized quantitative radiomics for high-throughput image-based phenotyping. Radiology:191145 10.1148/radiol.2020191145 10.1148/radiol.2020191145 PMC7193906 32154773

[130] Lambin P, Leijenaar RTH, Deist TM (2017). Radiomics: the bridge between medical imaging and personalized medicine. Nat Rev Clin Oncol.

[131] Park JE, Kim D, Kim HS (2020). Quality of science and reporting of radiomics in oncologic studies: room for improvement according to radiomics quality score and TRIPOD statement. Eur Radiol.

